# Cognitive Load and Self-Determination Theories Applied to E-Learning: Impact on Students' Participation and Academic Performance

**DOI:** 10.1371/journal.pone.0152462

**Published:** 2016-03-31

**Authors:** Tiago de Araujo Guerra Grangeia, Bruno de Jorge, Daniel Franci, Thiago Martins Santos, Maria Silvia Vellutini Setubal, Marcelo Schweller, Marco Antonio de Carvalho-Filho

**Affiliations:** 1 Department of Internal Medicine, Division of Emergency Medicine, Faculty of Medical Sciences at State University of Campinas (Unicamp), São Paulo, Brazil; 2 Faculty of Medical Sciences at State University of Campinas (Unicamp), São Paulo, Brazil; Kyoto University, JAPAN

## Abstract

**Background:**

Emergency clerkships expose students to a stressful environment that require multiple tasks, which may have a direct impact on cognitive load and motivation for learning. To address this challenge, Cognitive Load Theory and Self Determination Theory provided the conceptual frameworks to the development of a Moodle-based online Emergency Medicine course, inspired by real clinical cases.

**Methods:**

Three consecutive classes (2013–2015) of sixth-year medical students (n = 304) participated in the course, during a curricular and essentially practical emergency rotation. “Virtual Rounds” provided weekly virtual patients in narrative format and meaningful schemata to chief complaints, in order to simulate real rounds at Emergency Unit. Additional activities such as Extreme Decisions, Emergency Quiz and Electrocardiographic challenge offered different views of emergency care. Authors assessed student´s participation and its correlation with their academic performance. A survey evaluated students´ opinions. Students graduating in 2015 answered an online questionnaire to investigate cognitive load and motivation.

**Results:**

Each student produced 1965 pageviews and spent 72 hours logged on. Although Clinical Emergency rotation has two months long, students accessed the online course during an average of 5.3 months. Virtual Rounds was the most accessed activity, and there was positive correlations between the number of hours logged on the platform and final grades on Emergency Medicine. Over 90% of students felt an improvement in their clinical reasoning and considered themselves better prepared for rendering Emergency care. Considering a Likert scale from 1 (minimum load) to 7 (maximum load), the scores for total cognitive load were 4.79±2.2 for Virtual Rounds and 5.56±1.96 for real medical rounds(p<0,01).

**Conclusions:**

A real-world inspired online course, based on cognitive and motivational conceptual frameworks, seems to be a strong tool to engage students in learning. It may support them to manage the cognitive challenges involved in clinical care and increase their motivation for learning.

## Introduction

Students learn to be real doctors in the clinical environment, which is at the same time stimulating and challenging. In this setting, unanticipated events, patients too sick to participate in the teaching encounter and the difficulty to engage all learners simultaneously may affect teaching and learning [1,2]. Emergency Medicine environment takes those challenges to a higher level, as it deals with many complex diseases and demands quick decisions, good communication skills, and positive leadership in a multi-professional team [3,4].

Furthermore, undergraduate students are rarely in charge of patient care, which may cause distress and confidence reduction [4]. Noteworthy, it is hard to gather all students for a traditional lecture [5]. In this context, it is difficult to manage the cognitive load required to perform tasks adequately. As students have to deal with so many challenges, it may be hard to develop sustained relationships among them, with their teachers and patients, hindering intrinsic motivation for learning [6,7].

In order to deal with these challenges, teachers are required to have an expanded toolkit of teaching skills, taking into account conceptual frameworks [2]. They should deliver educational content in a way that facilitates students’ learning processes, and should support students to manage the clinical and emotional challenges involved in patient care [8].

Cognitive Load Theory (CLT) is a conceptual framework relevant to activities that involve executing tasks, by focusing on the management of working memory during learning. This framework describes the three aspects of cognitive load: intrinsic, extraneous and germane. The *intrinsic load* is related to the task difficulty and the learner’s level of expertise. The *extraneous* load is related to how the task is presented and to elements that are unconnected to the goals of the task and not essential for learning. The *germane* load is related to the learner´s level of concentration, which is important to long-term storage of new information [9,10,11]. This framework hypothesizes that a high extraneous load may jeopardize both intrinsic and germane loads on the working memory, hindering opportunities for effective learning [9,10]. So, an efficient instructional design should aim to minimize extraneous load, to manage intrinsic load and to optimize germane load [9,10,11].

Extraneous load may be minimized by reducing the ineffective load through diminishing the effect of environment on learning. Different pedagogic approaches can also reduce the extraneous load, such as, splitting the main task in partial ones which students can complete by themselves (problem completion), providing worked examples and maintaining one integrated source of information [10,12].

Intrinsic load should be managed through selection of tasks that matches learner´s level of expertise [13]. Germane load could be improved with activities that provide schemata construction and automation [9,10,12].

Total cognitive load have been measured with self-rating evaluation of learner’s mental effort, response time to secondary tasks, and psychophysiological measures [9,11]. Instruments such as the Paas Cognitive Load Scale [14] and the NASA Task Load Index [15] have been used as measures of total cognitive load [10].

Instructional designs that deal with the mental load will only be effective if students are motivated and ready to invest mental effort in it [16]. Therefore, motivation is another essential attribute of effective learning, and its influence has been described by studies in the field of psychology. According to the Self Determination Theory (SDT), the internalization process (from external regulation of behavior to self-regulation) may improve motivation for learning. It may be facilitated by accomplishing three innate psychological needs: the need for autonomy, competence and relatedness to others [6,7].

Active teaching methods are at least as effective as traditional techniques based on lectures [17–19], encouraging critical thinking, reflection and the development of clinical reasoning [20–24]. A variety of techniques has been included in the medical curriculum, such as high-fidelity simulation, problem-based learning, and distance education, especially over the Internet (e-learning) through virtual learning environments (VLE) [25].

E-learning offers countless advantages, such as its continuously available and updated content, which is easily accessed by a large number of students from anywhere, even through mobile devices [26,27]. It is possible to deliver knowledge through several different formats such as discussion forums, blogs, quizzes, hyperlinks, chats and wikis [5]. Interactivity may improve satisfaction and motivation for learning [28,29].

Therefore, e-learning has been widely used in medical education, usually in association with face-to-face activities, an approach known as blended learning. Although many studies show that students may be satisfied with e-learning [3], the perceptions and experiences of the students themselves have not been adequately addressed [29]. Besides, the impact of e-learning teaching strategies on students’ academic performance is poorly known [30–32].

We have developed an online Clinical Emergencies course on Moodle platform based on these two conceptual frameworks, CLT and SDT, aiming to enhance and facilitate student´s learning. Real clinical cases provided inspiration for our virtual patients, which were presented to students in a narrative format. Differently from traditional setups, in Virtual Rounds, we offered a step-by-step daily discussion in online forums during the course of a week, stimulating students to think about how he or she would proceed if they were in a real medical round. We posted a new case weekly over the course of three years, and used schemata (illness scripts) to foster the discussion of every common complaint that brings patients to emergency rooms. Some other VLE tools, such as diagnostic challenges based on supplementary tests (laboratory and imaging) and quizzes could sustain students’ interest in the online course and optimize their virtual experiences.

The purpose of this study was to assess sixth-year undergraduate medical students for their participation in and acceptance of this new learning environment, and to search for correlation between their participation and academic performance. We also measured the cognitive load related to our course’s tasks and students’ motivation for learning.

## Methods

### Participants

The subjects enrolled in our study were every sixth-year undergraduate medical students of three consecutive classes (2013–2015) at the University of Campinas. Students of the classes of 2013 and 2014 (n = 204) provided data related to participation and satisfaction in our course, and academic performance. Students graduating in 2015 (n = 59) answered an online questionnaire to investigate cognitive load and motivation.

### The Clinical Emergency Rotation

Clinical Emergency is a curricular and mandatory rotation that takes place in the sixth-year of our internship, lasting two months. In this rotation, all students spend one month at the Emergency Ward (EW) and the Intensive Care Unit (ICU), and another month at the Emergency Room (ER). They have practical supervised activities for 11 hours daily, Monday through Friday.

In the EW and ICU, students participate actively in daily bedside medical rounds, contributing with data that will be truly considered in the medical decisions related to patient care. Five physicians supervise students continuously, being responsible for in-person daily learning activities (Clinical Ethics meetings, Clinical Cases and Scientific Papers discussions).

At the ER, students perform supervised emergency care consultations, undertake procedures, shadow resident physicians and assistants, and participate in daily medical rounds with two instructing physicians. They also take part in weekly emergency care simulations using a high-fidelity mannequin (SimMan).

Student’s participation in the online course was voluntary and not graded. Their participation started from the beginning of their Emergency rotation period, and they were allowed to continue using the platform until they want. Other activities in the Emergency Discipline such as Clinical Ethics Meetings, Clinical Cases Discussions, Scientific Papers Discussions and Simulations were all mandatory.

### Content of the online course

An ER instructing physician who is with the students on daily basis was in charge of creating the VLE course content, based on real clinical cases seen in the ER and EW/ICU at least three months before. Our main purpose was to allow students to experience the decision-making process at online course. They could assume the role of the leading physician and draw a direct relationship between the virtual environment content and its application in actual care.

Our main activity was **Virtual Rounds**, an online-shared forum among students and teachers. Our main objective was to reproduce a real bedside medical round in the virtual environment. The discussions started with the chief complaint, encouraging students to deal with the differential diagnosis and to establish a treatment plan. They would answer open-ended questions posted by the teacher in a narrative format, and share their thoughts with the group. In this way, students could have a glimpse of what happens in the physician´s mind throughout the diagnostic process, and even discuss with peers and teachers specific points that are often omitted during a real medical round. Usually, students have no access to the decision-making process as a whole, and the virtual environment may provide adequate time and availability for this to occur.

On Virtual Rounds, each case has five parts, one for each day of the week, compelling students to reflect on their medical actions every day. On Monday, we posted the first part of the case, prioritizing the chief complaint that had led the patient to the emergency room (e.g., headache, chest pain, respiratory distress, coma). Then, students answer open-ended questions designed to extract the most important data from the clinical history and physical examination.

For each subsequent day (Tuesday through Thursday), those questions were answered, new information was provided (including figures, tables and imaging tests), and new questions were posted. The purpose was to discuss differential diagnosis and to establish a treatment plan. On Friday, the teacher presented the patients’ outcomes. In this way, students were able to face simple-to-complex sequencing of questions based on authentic, real-life tasks, following the same steps necessary in the real world. In addition, students could compare their peers and teachers’ thoughts with their own, since answers remained available at the forums.

In three years, we posted 144 cases, providing variability of practice. Every case remained available throughout the whole course, being studied as worked-examples. These cases were also categorized and grouped according to the chief complaint, and students could choose to study randomly or focused on a topic. After participating in the week’s Virtual Round, students could compare the present case with previous ones, especially those with the same chief complaint but diverse final diagnosis, progression and outcomes. Students’ average time to complete a Virtual Rounds case was around 10 minutes per day.

#### Schemata and Virtual Rounds case construction

According to the Cognitive Load Theory, the most important learning processes for developing the ability to acquire knowledge and skills are schemata construction and automation [9, 10, 16].

Each external stimulus, such as sounds and images, enters the brain as a single unit of information, primarily through the working memory (WM). WM may process up to 9 units at the same time, being easily overloaded when it is dealing with a novel task that requires a large number of information units [33, 34].

When the student dedicates attention to the external information, multiple units can be grouped into “chunks” that will be stored as a single unit at the long term memory (LTM), providing a schema for the studied task. Therefore, schemata organize knowledge and markedly reduce WM load, because even a complex schema can be recovered as one unit of information [35].

Besides, information is retained by the WM for a limited period of time, up to 30 seconds. For this reason, it is frequently necessary to rehearse by repetition to retain information and store it in the LTM [10].

In Virtual Rounds, we used evidence-based Medicine to create schemata. For each main complaint that may drive a patient to the emergency department, we offered the signs and symptoms that increase or reduce the probability of each differential diagnostic. Therefore, each major clinical complaint had an individual schema ("illness script") which would be repeated in subsequent cases that had the same initial complaint. Each schema had sequential steps to help students to manage their clinical reasoning. Our aim was to provide schema automation for recurrent aspects of the whole task, trying to improve student´s clinical reasoning. In Table 1 we present initial complaints that had their individual schema on our online course.

**Table 1:** Initial complaints that had their individual schema.

Chest Pain	Diarrhea	Syncope
Sepsis	Headache	Low back pain
Shock	Sore throat	Delirium
Respiratory Failure	Abdominal Pain	Palpitations
Dyspnea	Cough	Jaundice
Coma	Hemoptysis	Lower limb edema

**Fig 1:**
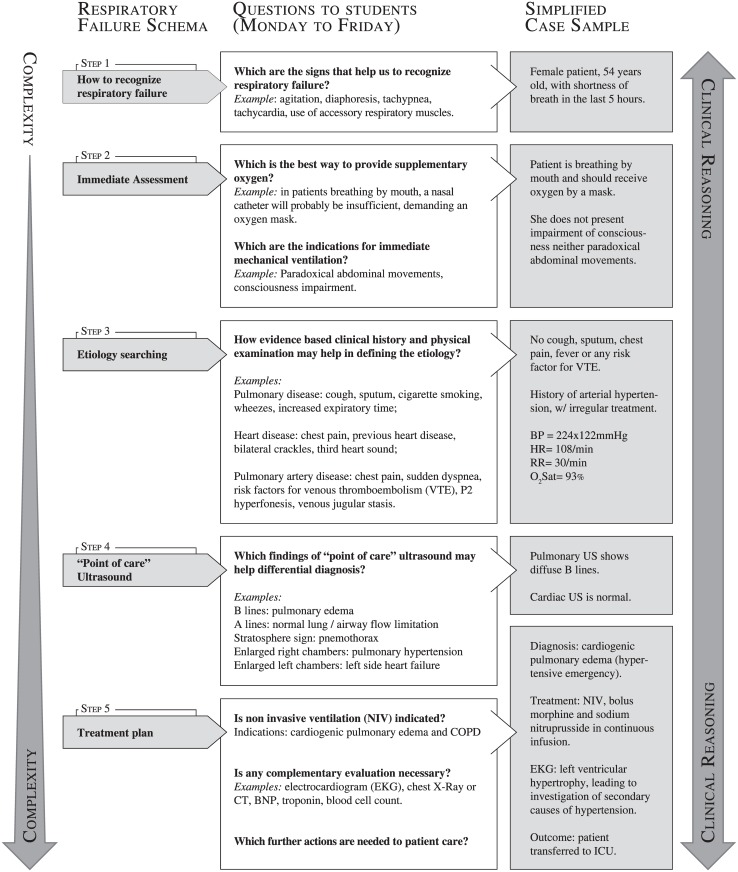
Virtual Rounds case construction—Respiratory Failure. At left, a schema is shown in six successive steps, discussed during the week. In general, one or two steps are discussed in a single day, from Monday to Friday. The following steps were addressed only after the previous had been accessed. At right, we provide a resumed Virtual Round model, with the main information that would be discussed at the online shared forums with students, having the schema as a guide.

**Fig 2:**
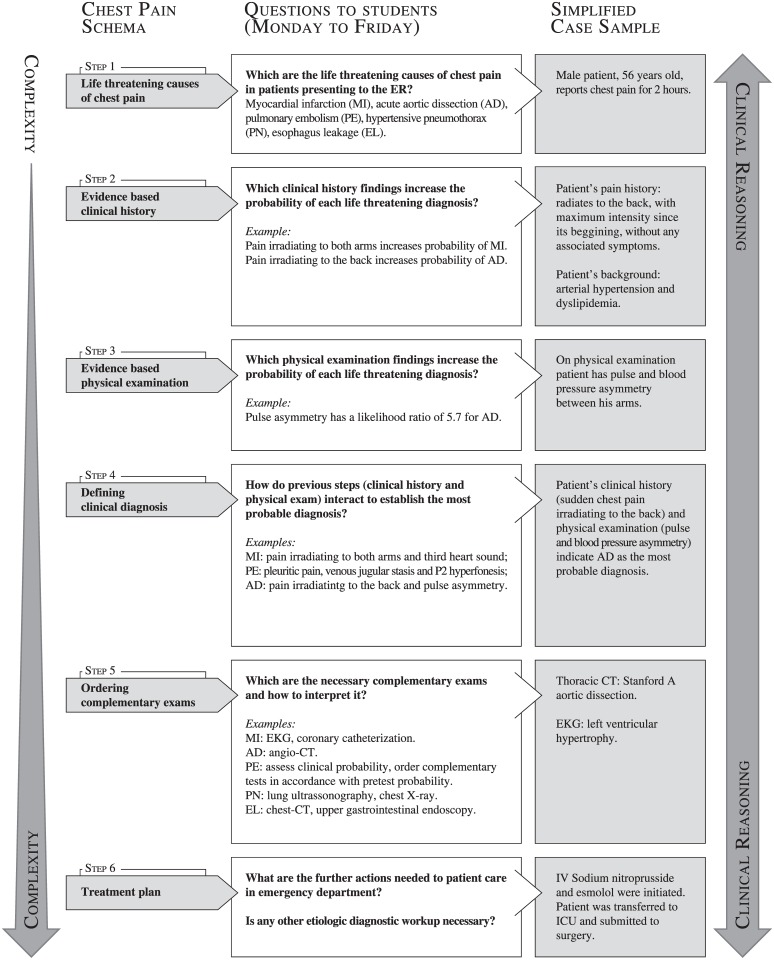
Virtual Rounds case construction—Chest Pain. At left, a schema is shown in six successive steps, discussed during the week. In general, one or two steps are discussed in a single day, from Monday to Friday. The following steps were addressed only after the previous had been accessed. At right, we provide a resumed Virtual Round model, with the main information that would be discussed at the online shared forums with students, having the schema as a guide.

**Fig 3:**
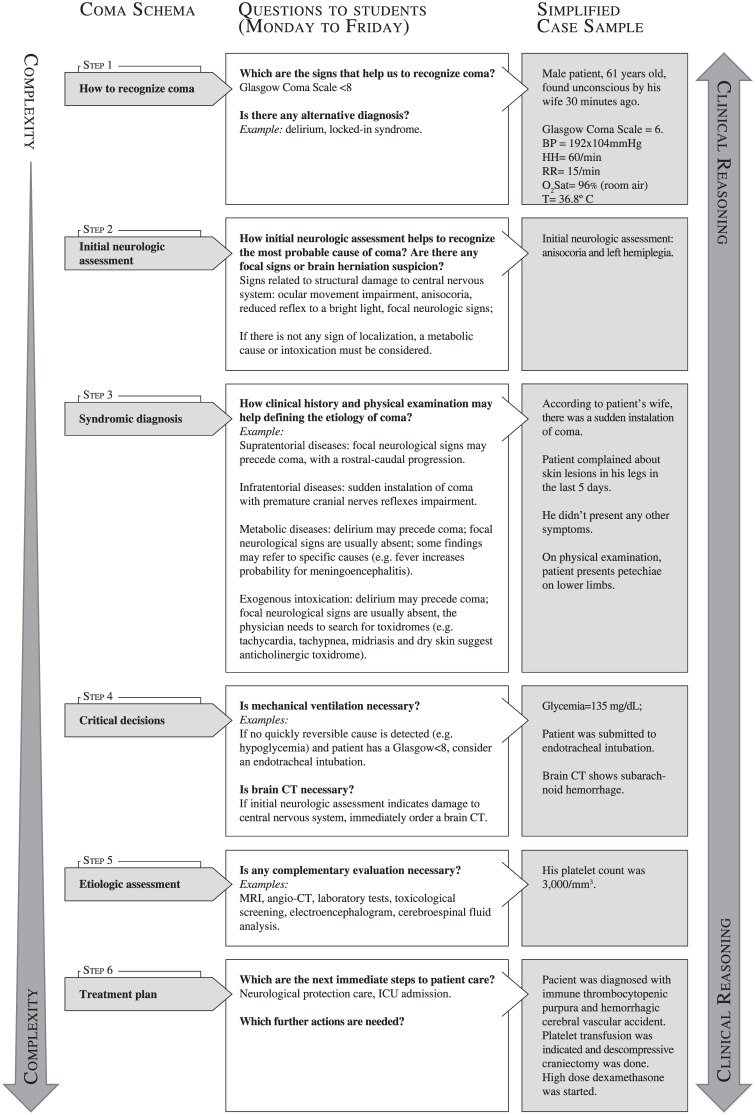
Virtual Rounds case construction—Coma. At left, a schema is shown in six successive steps, discussed during the week. In general, one or two steps are discussed in a single day, from Monday to Friday. The following steps were addressed only after the previous had been accessed. At right, we provide a resumed Virtual Round model, with the main information that would be discussed at the online shared forums with students, having the schema as a guide.

Schemata were structured by a series of clinical problems of increasing complexity, and each one of these problems can only be solved when the previous one is adequately addressed. Every clinical case that starts from the same initial main complaint is discussed following the same clinical reasoning steps based on the specific schema for that complaint. Repetition may help students to internalize the clinical reasoning for each complaint. Figs 1–3 show simplified diagrams of schemata used by teachers to elaborate Virtual Rounds’ clinical cases.

#### Other VLE activities

Other VLE activities were also available, with the intention of stimulating students’ interest on the course and providing theoretical background relevant for the solution of Virtual Rounds cases. These activities are presented below.

#### Emergency Quiz

Two hundred multiple-choice questions were available. Students received immediate feedback soon after choosing an alternative whether correct or not. Each Quiz could be accomplished in an average time of 10 minutes.

#### Extreme Decisions

Weekly virtual patients based on real ER cases, focusing on quick decision-making situations, such as an asthma exacerbation, from admission to discharge. These cases were presented through sequential multiple-choice questions (eight to twelve), revisiting the steps a real doctor should follow. Immediate feedback occurred upon clicking on an alternative, whether correct or not. Each Extreme Decision could be accomplished in an average time of 15 minutes. We created thirty cases in this format.

#### Electrocardiogram and Radiology Challenges

Weekly clinical case discussions assessing supplementary tests essential to the case diagnosis and treatment plan, placing the student in the decision maker position. We created 120 challenges for each format throughout the years. Each one of these Challenges could be completed in an average time of 10 minutes.

#### Links to related bibliography

We have posted hyperlinks to review papers related to each activity for every Virtual Round, Emergency Quiz and Extreme Decisions. So, students could access papers directly from the activity of interest, without being necessary looking for other sources. We have tried to avoid split-attention, providing all information in integrated way.

### Feedback

In Virtual Rounds, the teacher provided answers for the questions posted the day before, and for additional questions asked by students. The main purpose of this specific teacher intervention was to promote cognitive and friendly feedback on a daily basis. Students could reflect beyond teacher´s and peer´s answers and post their own conclusions.

Students also received teacher’s feedback regarding Electrocardiogram and Radiology challenges within a week. Activities with multiple-choice questions had immediate and automatic feedback. In Extreme Decisions, not only the students had immediate feedback, but were also invited to post specific questions, answered through personal e-mail within 48 hours.

### Tutor´s work

The tutor dedicated at least 20 hours per week to the online course activities, including creating course content, providing feedback and analyzing students’ participation. In order to provide feedback, the tutor had to look for answers and doubts of students every day, at least one time. Some activities, such as, Emergency Quiz and Extreme Decisions had a previous set, automatic feedback. An educational web designer helped the tutor posting the content in the Moodle platform.

### Assessment Methods

Along the two months of Emergency Medicine rotation, every student took two theoretical tests and two practical tests (OSCE format), one midterm and one final. The weighted average of these exams (60% for the theoretical exam and 40% for OSCE) comprised their final grade. If the student had an insufficient score, a third exam was applied at the end of the two months period. At the end, all the students were approved in the rotation. It is worth knowing that all the exams were performed during the rotation, which means that at the end of the rotation all students were aware about their final grades.

In each rotation site (EW/ICU and ER), preceptors performed the attitudes and behaviors assessment (AAB), including technical aspects (clinical history and the physical exam), attitudes (willingness to learn, medical visit and chart organization) and behavior (towards the medical team, patients, his/her family and health team). Three real patients also graded students’ performances right after their consultation using the CARE (Consultation and Relational Empathy) scale.

### Student satisfaction survey

At the end of the Emergency rotation, students of 2013 and 2014 academic years filled out a survey posted on the Moodle platform to assess their impressions about the course. The answers could be “yes”, “no” and “indifferent”. A text box for each question allowed students to further elaborate on their answers and write comments about their experience of participating in VLE activities. The questions were: (1) “Which category do you identify as the one that most contributed to your learning? Why?” (2) “Did the Virtual Rounds cases stimulate your clinical reasoning while seeing patients in the emergency room? How?” (3) “Did the information and discussions supplied in the Virtual Rounds stimulate you to increase your participation in real medical rounds in the emergency room? Why?” (4) “Did the discussions of diagnostic tests (electrocardiogram and radiology) in the form of weekly challenges help you interpret these exams when seeing a patient in the emergency room? How?” (5) “Did you feel stimulated to read the review papers listed in the bibliography? Why?” and (6) “After your participation in the course, do you feel better prepared to render emergency care? Why?”

### Measurement of Cognitive Load

At the end of Emergency Rotation in 2015, we have measured the total cognitive load through a self-reported questionnaire based on the scale developed by Pass [36]. It is a 7-point Likert scale (1 = minimum; 7 = maximum).

The first two items of the questionnaire addressed total cognitive load of virtual and real rounds:*“How much mental effort was necessary to accomplish Virtual Rounds*?*”* and *“How much mental effort was necessary to accomplish real medical rounds*?*”*

Additional items addressed specific aspects of cognitive load on our online course: *“How difficult was the online course content*?*”* (intrinsic load); *“How difficult it was to navigate on our platform*?*”* (extrinsic load); *“How much knowledge did you acquire after participating in our online course*?*”* and *“How much concentration did you maintain during our course activities*?*”* (germane load). We also made a question about motivation: *“How motivated were you to participate in the online course*?*”*. Students were asked to name three main reasons to their motivation.

### Statistical analysis

We provide a *descriptive analysis* of students’ access and participation in the online course.

We looked for correlations between students’ participation in our course and their academic performance. We used Spearman’s analysis to correlate *the number of hours logged on* during Emergency Rotation with *academic performance*, through 1) the final grade in the Clinical Emergency Discipline, 2) the theoretical exam, 3) the OSCE, 4) the CARE scores, and 5) the AAB assessment. For this purpose, it is worth registering that only the students’ online production that happened during the two months period of their rotation were utilized in correlation analysis. The student’s online production after the end of the rotation was not used in statistics analysis of academic performance, since it would not have any impact in students’ emergency rotation grades.

We also ran ordinal logistic regression models to analyze the relationship between the grade (divided in four quartiles) and the hours logged on the course.

To statistical analysis, we used the SAS System for Windows (Statistical Analysis System), version 9,4. SAS Institute Inc, Cary, NC, USA.

#### Surveys Analysis

We analyzed students’ answers to the questionnaire of Student Satisfaction Survey. Results were described in percentages. The content of the open-ended questions underwent thematic content analysis after exhaustible material reading. We came up with hypothesis and objectives and selected some responses for analysis to identify the most relevant categories for each topic, which we transcribed exactly as written by students.

In order to measure the cognitive load related to Virtual Rounds and real medical rounds, we analyzed the answers of sixth-year students of the 2015 academic year. After defining that the answers had a normal (Gaussian) distribution, we performed a t-test for paired samples to compare the students’ cognitive load on both environments. The remaining items of the questionnaire underwent a descriptive analysis.

Students provided three main reasons that motivate their participation in our course. The most frequent answers were grouped at a word cloud, appearing bigger on the final diagram.

### Ethics Statement

The “Comitê de Ética e Pesquisa da Unicamp”, that is the Ethics committee on human beings from the School of Medical Sciences of the University of Campinas (Unicamp), judged that it was not necessary to get written informed consent of the participants in this study. The main reasons were: our course was already curricular at that point; we did not used any data that allowed student´s individual identification.

The study was approved by the Ethics committee. Number of approval: 451,130 (CAAE 20424013.1.0000.5404).

## Results

Among 204 students from 2013 and 2014 academic years, 92 (45.1%) were male and 112 (54.9%) were female.

All students accessed the platform during the Emergency rotation period. Table 2 displays the data from student’s general participation (number of hours logged on, and total numbers of pageviews, accesses and of posts), as well as the means per student, considering the rotation period and the entire academic year.

**Table 2:** Student participation at the emergency VLE course.

	Rotation Period	Total	Mean per student
**Hours (h)**	13,289	14,689	71.9
**Pageviews**	367,775	400,932	1,965
**Accesses**	9,701	10,720	49.4
**Posts**	3,174	3,450	17.1

General access figures for the VLE course; sixth-year students of academic years 2013 and 2014 (n = 204).

One hour and 39 minutes was the average time a student remained logged on to the platform during each access event.

Table 3 shows the percentage hours spent logged on for each online activity, with Virtual Rounds being the most accessed.

**Table 3:** Proportional students´ participation on each activity.

Activity	Percentage of hours logged on(%)
Virtual Rounds	44.5
Extreme Decisions	21.1
Emergency Quiz	21
Electrocardiogram Challenges	8.5
Radiology Challenges	4.9

Hours spent logged on, in percentages, by activity.

Although the Clinical Emergencies rotation is two months long, students accessed the online course during an average of 5.3 months, reflecting the fact that 76% of students also accessed the platform outside the Emergency Rotation period.

**Fig 4:**
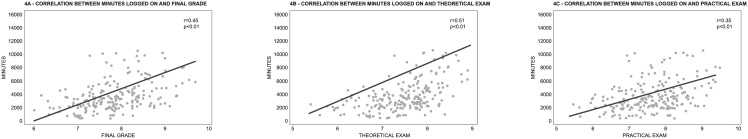
Correlations between number of hours logged on during the rotation period and final grade. **4A)** Correlation between the number of hours logged per student during the rotation period and final grade in the discipline–Spearman coefficient = 0.45 (p<0.01); **4B)** Correlation between the number of hours logged on during the rotation period and the grade on the theory exam–Spearman coefficient = 0.51 (p<0.01); **4C)** Correlation between the number of hours logged on during the rotation period and the score on the practical exam—Spearman coefficient = 0.35 (p<0.01).

There was a positive correlation between the number of hours the student was logged on during the rotation period and their final grade (r = 0.45; p< 0.001)–Fig 4a. We observed a positive correlation between the number of hours logged on during the rotation period and the score on the theoretical exam (r = 0.51; p< 0.001)–Fig 4b, and between the number of hours logged on during the rotation period and the practical exam (r = 0.35; p = 0.01)–Fig 4c. No correlation was found between the number of hours logged on during the rotation period and the patient evaluation scores (r = 0.08; p = 0.4).

A weak positive correlation was found between the number of hours logged on during the rotation period and the AAB in the ER (r = 0.18; p = 0.009), and in the EW/ICU (r = 0.13, p = 0.05).

**Fig 5:**
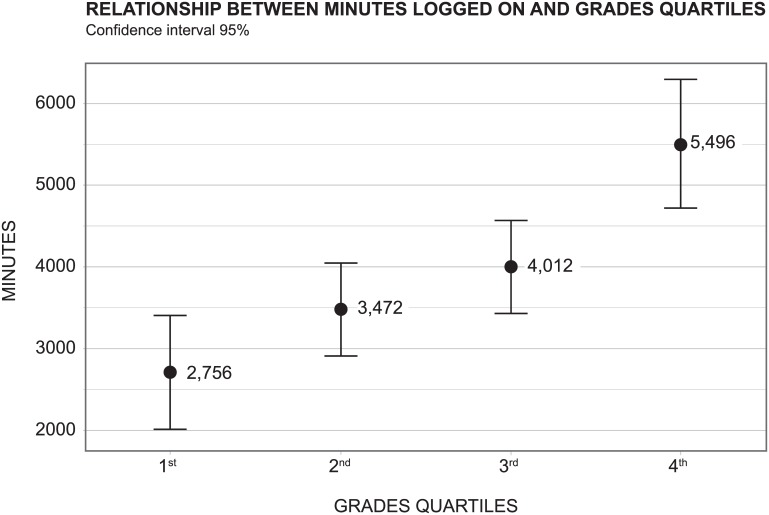
Confidence interval between minutes logged on during the rotation period and final grade. Confidence interval between minutes logged on during the rotation period and final grade (divided in quartiles).

Students were divided in quartiles according to their grades in Emergency Medicine (1^st^ being the lowest and 4^th^ the highest). Fig 5 shows the confidence interval (95%) relating to minutes logged on during the rotation period and the grades quartiles. The more time students spent on the Moodle platform (in mean and standard deviation), the higher the quartile they got into.

Table 4 shows that when students spend more time logged on during the rotation period, their probability of being in better quartile of grades increases. For example, for each extra 60 minutes logged on the platform during the rotation period there is a 3.3% higher chance for a student to be in the 4^th^ quartile of grades.

**Table 4:** Number of minutes logged on during the rotation period and the probability of being in better grades quartiles.

Quartiles	Odds ratio	Each extra 60 min logged on	P—value
**2**^**nd**^ **x 1**^**st**^	1.0002	1.014	0.0440
**3**^**rd**^ **x 1**^**st**^	1.0003	1.021	0.0031
**4**^**th**^ **x 1**^**st**^	1.0005	1.033	< 0.001

Number of minutes logged on during the rotation period and the probability of being in better grades quartiles.

All data produced by students are available in Supporting Information files. S1, S2 and S3 Files contain all logs (pageviews) produced by students, during and outside emergency rotation period. S4 File contains Student´s Grades.

### Answers to the satisfaction survey

Among 204 students of academic years 2013 and 2014, 123 (60.2%) filled out the satisfaction survey. Virtual Rounds was considered the activity that contributed the most to their learning for 66% of the group.

The answers to the first question revealed that 97% of the respondents reported that Virtual Rounds stimulated their clinical reasoning in emergency care, and 82% reported that it encouraged them to increase their participation in real medical rounds. The main reasons given by students were, in rank order, (1) systematization of clinical reasoning, (2) structuring of learning materials (common complaints, syndromic diagnosis and patient evolution), (3) gaining of knowledge and (4) confidence. Some statements represent these opinions:

“*Virtual Round made me reflect on how to reach the patient’s diagnosis*. *It encouraged me to think about differential diagnosis and it gave me more tools to do so*. *Besides*, *it is so similar to real ER cases that I felt more capable to deal with doctor’s day-to-day life*”.

*“When you come in contact with your colleagues’ opinions about the topic*, *and later with the answers to their questions*, *you feel more comfortable to discuss it in a real medical encounter*. *It brings a greater sense of confidence to assess and discuss the cases…*.*”*.

Some answers related to the Virtual Rounds also shows how some students perceive the differences between the cognitive load of the “real world” and the “virtual world”:

*“Patients usually stay at the ER for a short period of time*, *being difficult to develop a clear line of thought about his or her history and diagnosis*. *Virtual Rounds provided a systematization of the clinical reasoning*, *helping me to understand the cases*.*”*

Forum discussions regarding diagnostic tests helped 97% of respondents in interpreting them by allowing (1) the recognition of patterns and (2) the organization of the reasoning behind them. A particular response to exemplify it was:

*“They increased my ability to analyze these tests*. *The way in which these challenges are answered helped me to learn more about them*. *I was able to interpret these tests before but the course gave me the ability to organize my reasoning*, *standardizing the way to analyze them*, *which made me get much more information than I was able to before”*.

The great majority of respondents (93%) felt better prepared for rendering emergency care after participating in the course, mainly due to, in rank order, (1) the systematization of knowledge, (2) greater confidence, (3) the fact that the content reflected reality and (4) the strong theoretical substrate. We identified the following responses that exemplify this finding:

*“The course is complete and very close to the reality*. *I never found anything that helped me learn in such a real and practical manner”*.

*“The course was based on cases that I encountered in my emergency internship*. *The virtual activities expanded my knowledge of basic emergency situations*. *It was easy for me to look up the website after any case*. *Most importantly*, *I first saw some cases in the course and then in real life*, *which helped me to better address the real case”*.

*“Despite the lack of confidence for being on my own to assess a case from now on*, *the course helped me by showing the steps to be followed*, *approaching the signs and symptoms*. *It was not like the textbooks that just show the previously diagnosed disease*. *This approach is much closer to our reality and that is why the course will always be a great study source for me”*.

Some statements show the relation between the online course and students’ motivation:

*“After studying and researching to answer Virtual Rounds*, *I developed the habit to solve clinical cases*, *virtual or real*. *When facing a real case in which the diagnosis is not evident*, *I found myself willing to solve it*, *instead of waiting for the teacher to give me the solution*.*”*

### Measurement of cognitive load

Among 100 students of 2015 academic year, 59 filled out the questionnaire to assess cognitive load. Considering a Likert scale from 1 (minimum load) to 7 (maximum load), the scores and standard deviations for total cognitive load were 4.79 ± 2.2 for Virtual Rounds and 5.56 ± 1.96 for real medical rounds (p<0,01).

The difficulty of the online content received a score on Likert scale (1–7) of 4.35 ± 1.32; the difficulty to navigate on the platform had a score of 1.74 ± 1.05; the amount of knowledge acquired with the course received a score of 6.32 ± 0.70; and concentration achieved a score of 6 ± 1.18.

When asked to grade their motivation to participate in the online course, using a similar Likert scale from 1 (no motivation) to 7 (maximum motivation), score was 6 ± 1.15.

**Fig 6:**
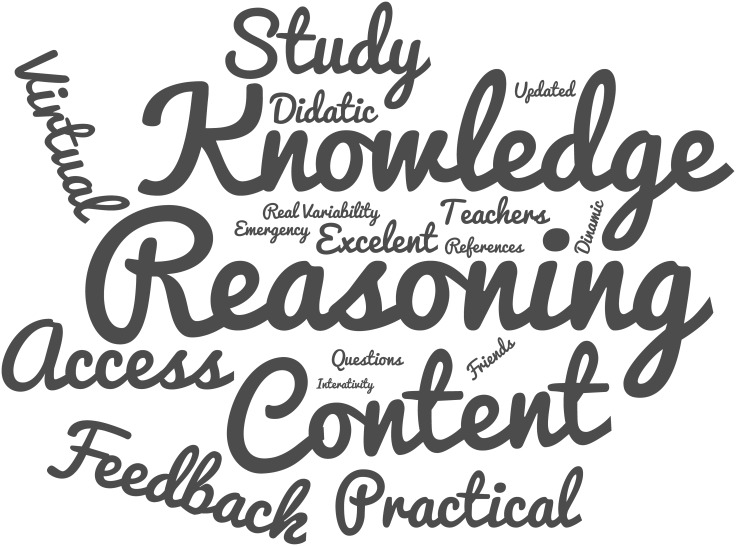
Reasons that motivated students to participate on our online course. Word cloud presenting the reasons that motivated students to participate on our online course.

Fig 6 shows the main reasons that motivated students to participate on our online course, grouped in a word cloud.

## Discussion

In order to address the multi-challenged Emergency Medicine learning environment, we offered a Moodle-based course with online didactic activities inspired by real clinical situations. Sixth-year undergraduate medical students dedicated several hours studying the platform content and used it during and after their Clinical Emergencies rotation. Their academic grades were somehow correlated with the intensity of their platform use.

Students’ acceptance and satisfaction may be related, at first, with the fact that they are continuously connected to the internet and enjoy being able to study whenever and wherever they want. Besides, the course was based in the Cognitive Load Theory (CLT) and the Self Determination Theory (SDT), two conceptual frameworks that may have facilitated and optimized students’ learning experiences and their academic performance [6,7,9,10].

CLT is relevant to activities that involve executing tasks, by focusing on the management of working memory during learning, especially through two major processes: schemata construction and automation. In this sense, three strategies may facilitate learning: decrease of extraneous load, management of intrinsic load and optimization of germane load [9,10].

Extraneous load encompasses everything that is not essential to the task, and may jeopardize schemata construction and automation [9,10]. In emergency department, student’s anxiety, self-consciousness at being observed by an attending, fatigue, distractions and complex diseases may increase extraneous load and ‘consume’ working memory resources [37]. At our course, students could access the platform wherever they want, minimizing learning interruptions and the impact of environment on learning (multi-professional team interactions, crowded departments). Also, in Virtual Rounds we tried to reduce extraneous load by inviting students to complete a clinical problem in sequential days, taking the steps that were missing the day before, a technique known as problem completion. Finally, every case of Virtual Rounds activity remained available throughout the whole course, being studied as worked-examples by students.

We managed intrinsic load gradually increasing the amount of information and the complexity of the case through the week, in our Virtual Rounds category. Van Merrienboer and Sweller [38] consider approaches like “simple-to-complex” fully in line with cognitive load theory, as they start with few elements and gradually build up complexity [39]. In our course, each day students had to face activities that were more complex when compared with activities from the previous day. Students could activate prior knowledge daily, contrasting it to new information, in order to elaborate further knowledge.

Finally, germane load is the mental effort needed for learning [9,10]. It could be optimized by increasing the diversity of available tasks (discussion forums, quizzes, lessons, hyperlinks), and by stimulating students to imagine themselves solving those clinical problems. Beyond that, strategies like schemata creation and automation allows deep learning and systematization of knowledge. We provided schemata for every chief complaint (schemata creation) that were repeated in every case with the same chief complaint (automation).

Repetition and rehearsal may lead schemata to become automated. Information is then organized without excessive burden to working memory [9,10]. In our online course, Virtual Rounds category offered 144 cases, discussed following all the steps that would be necessary in the real world. The schemata directed to the initial chief complaint would be repeated in all cases that started with the same complaint. Of the respondents to our survey, 66% named the Virtual Rounds as the activity that contributed the most to their learning.

All these strategies were reflected in student´s answers to our questionnaire to measure cognitive load. They provide small score for extraneous load and high scores for germane load.

In addition, motivation and emotion also influence learning and performance [6,7,9]. We looked for strategies to increase the intrinsic motivation of our students to learn Emergency. According to SDT, the internalization process (from the external regulation of behavior to self-regulation) may improve motivation for learning and could be facilitated by accomplishing three needs: autonomy, competence and relatedness to others [6,7].

Our students had autonomy to participate at our course, since it was not part of the summative assessment. They were also free to select the most relevant categories to them and formulated their own learning objectives. Each student spent, in average, almost 70 hours in our platform.

Adult learning theories frequently state the importance of the perception of practical application of knowledge in the learning process [40]. As students performed the activities in several categories, they compared new information with what they already knew, completed quizzes, and received feedback, which may have improved their sense of competence. As a blended learning strategy, students could apply the knowledge acquired online on their real-world activities the next day, further increasing the sense of competence. This idea became palpable when 93% reported to be better prepared for rendering emergency care.

Students could collaboratively work in problems through shared forums, interacting with their colleagues and being exposed to their doubts and difficulties in a protected way. Furthermore, the professors who created the VLE course content were the same ones guiding the students in their medical rounds during those two months. Teachers were able to demonstrate the course practical applications on a daily basis, thereby boosting students’ learning and knowledge retention [40]. In this sense, it was possible to address the need to relatedness, and 82% of respondents considered themselves better prepared to participate in the real daily medical rounds.

Students kept their interest in the Clinical Emergencies course during the internship and many months afterwards, an indirect evidence of motivation improvement. The mean time of platform use was 5.3 months, much longer than the two months allocated for the rotation. In this way, we could hypothesize that the senses of autonomy, competence and relatedness could promote self-regulation and intrinsic motivation for learning. Although some students may initially have participated in the online course concerned about tests and assessments (external regulation of behavior), they gained knowledge and progressively felt more competent. Their intrinsic motivation improved, and they were interested in acquiring and producing knowledge. Student´s answer to our questionnaire indicate high score to motivation, and they named the three main reasons to their motivation improvement: clinical reasoning, knowledge acquisition and clinical content.

Feedback is an essential feature of every educational strategy, including online courses [3,31,41] We have made feedback available to students immediately after their answers to multiple-choice questions and within 24 hours in Virtual Rounds discussion forums. It may have contributed to the course’s wide acceptance by students.

We found positive correlations between online participation (time spent logged on to the platform during rotation period) and academic performance (final grades in the Clinical Emergency discipline), showing that students who used the platform more diligently during the rotation scored better. There was a positive correlation between the number of hours logged on and the grades on exams, as we found that to every 60 min logged on to our platform, there was an increase of 3.3% in the student’s chance to be placed in the best grade’s quartile. We hypothesize that learning helps to develop a sense of confidence which evolves to better performance. However, we should be careful interpreting these data, since our investigation was not randomized and it is possible that students who performed better, were initially more motivated, and therefore more prone to use the platform. In that case, it could be just an attitude issue, and not a direct effect of platform use. Unfortunately, we have not had access to students’ performance in other disciplines to compare their grades and minimize this limitation.

A similar study based on virtual clinical cases did not find a positive correlation with exams [42]. There is a lack of definitive literature on the best ways to use e-learning, and its impact on academic performance [3,29–32,43].

Literature shows how difficult it is to build communication and empathy skills through virtual courses only [44–48]. We found a weak correlation between the number of hours students dedicated to the online course during the rotation and the attitudes and behaviors assessment (AAB) in the ER and EW/ICU. These grades reflected not only cognitive medical aspects but also attitudes and communication skills towards the patient and their family.

In Box 1 we summarize important aspects for the development of an online medical course based on these two conceptual frameworks.

**Box 1. Important aspects to the development of an online medical course based on CLT and SDT**

**Online course development**

Real clinical cases

Narrative format

Updated content

Continuous availability

**Cognitive Load Theory**

Schemata Construction

Automation

Simple-to-complex tasks sequencing

Problem completion

Worked examples

Reduced environment impact on learning

**Self Determination Theory**

Immediate feedback

Self-assessment questions

Shared forums

Same teacher in both environments (real and virtual)

Blended learning strategy

Our work has some limitations. There was no control group to compare academic performance. We did not use a specific tool to measure students´ participation during real medical rounds or to assess gains in clinical reasoning. Instead, we measured the correlations between the theoretical and practical exams with participation in the course during the rotation period. As we have commented, it is an indirect measure which must be cautiously considered, since it cannot definitively prove a causal relationship between online learning and performance.

Besides, students of academic years 2013 and 2014 provided data related to participation in our course, correlations with academic performance and answers to the satisfaction survey and the students of academic year 2015 answered a questionnaire to measure cognitive load and motivation related to our course.

## Conclusion

An online course, inspired in real patients and built in a narrative format that recreates every stage of emergency care, may be an effective way to provide students a theoretical basis in Emergency Medicine. The online course might also foster the confidence needed to adjust the learning process to their real experiences in the ER.

To assure effective learning in the clinical environment, it is fundamental to reduce the extraneous cognitive load, manage the intrinsic load and optimize germane load. When developing an online medical course, it is important to consider the cognitive load imposed to students, in order to attract their interest and stimulate their participation. It is also important to use strategies to increase their motivation for learning, ensuring that the need for autonomy, competence and relatedness to others would be contemplated. In this context, the conceptual frameworks CLT and SDT complement each other, guiding the online course development and facilitating students’ learning.

The following aspects may contribute to students’ acceptance of a VLE course: schemata, automation, immediate feedback, interaction among peers, use of several online tools, continuous course availability, and the perception of the immediate application of knowledge.

## Supporting Information

S1 File Student´s pageviews part I. Excel worksheet with logs produced by students. Legend: ID = each number represents an individual student; Action: URL of activity on our Moodle platform; Information: which specific activity was accessed by student; Category = which activity was accessed by student; Hour: time spent logged on; Rotation: during = log produced during emergency rotation period; outside: log produced outside emergency rotation period.
(XLSX)

S2 File Student´s pageviews part II. Excel worksheet with logs produced by students. Legend: ID = each number represents an individual student; Action: URL of activity on our Moodle platform; Information: which specific activity was accessed by student; Category = which activity was accessed by student; Hour: time spent logged on; Rotation: during = log produced during emergency rotation period; outside: log produced outside emergency rotation period.
(XLSX)

S3 File Student´s pageviews part III. Excel worksheet with logs produced by students. Legend: ID = each number represents an individual student; Action: URL of activity on our Moodle platform; Information: which specific activity was accessed by student; Category = which activity was accessed by student; Hour: time spent logged on; Rotation: during = log produced during emergency rotation period; outside: log produced outside emergency rotation period.
(XLSX)

S4 File Student´s grades in Emergency Rotation. Student´s grades in Emergency Rotation—final grades, theoretical exam and practical exam.
(XLSX)
